# P21 The fly in the ointment: a rare cause of cytopaenia

**DOI:** 10.1093/rap/rkac067.021

**Published:** 2022-09-28

**Authors:** Tegwen Ecclestone, Peter Merry

**Affiliations:** Rheumatology Department, Norfolk and Norwich University Hospital, Norwich, United Kingdom; Rheumatology Department, Norfolk and Norwich University Hospital, Norwich, United Kingdom

## Abstract

**Introduction/Background:**

Those working in Rheumatology frequently encounter cytopaenia both as adverse effects of many conventional and biological disease modifying drugs and as manifestations of autoimmune diseases. In cases where cytopaenia progresses, other explanations should be sought. Here we present an unusual cause of cytopaenia, seen in a patient with psoriatic arthritis on immunosuppression.

**Description/Method:**

An 81-year-old woman with a diagnosis of psoriatic arthritis had been maintained on Infliximab (3mg/kg 8 weekly) and Methotrexate (7.5mg PO weekly) for 12 years. She then developed mild neutropaenia (1.52 x 109/L). Methotrexate was stopped and she continued on Infliximab monotherapy for several months, until ongoing neutropenia resulted in Infliximab being withheld. Sustained mild neutropenia and the development of thrombocytopenia (platelets 95 x 109/L) prompted a referral to Haematology, who suspected autoimmune neutropenia and advised checking haematinics and monitoring the FBC monthly. The neutropenia improved and Infliximab was restarted.

Over 6 months she developed gradually worsening and persistent pancytopenia (neutrophils 1.36 x 109/L, platelets 134 x 109/L, Hb 91 g/L). There was no history of fevers, night sweats, bone pain, headaches, recurrent infections, lumps or bumps, chest symptoms, change in bowel habit or urinary symptoms. Examination revealed no pallor, jaundice, clubbing, peripheral lymphadenopathy or sternal tenderness. The chest was clear and the abdomen soft with no organomegaly.

Repeat bloods showed microcytic hypochromic anaemia (Hb 86 g/L, MCV 73 fL, MCH 24.6 pg) with target cells on blood film. Iron was low (iron 5.1 umol/L, transferrin 1.52 g/L). Serum protein electrophoresis showed paraprotein bands with abnormal serum free light chain assay possibly consistent with myeloma, lymphoma or MGUS. Haematology suspected myelodysplasia, potentially secondary to previous long term Methotrexate therapy. A bone marrow biopsy was performed; the unexpected result was Leishmaniasis. Our patient was admitted to hospital and, following microbiology review, was treated with liposomal amphotericin (IV) for Visceral Leishmaniasis as an inpatient. Her Infliximab was suspended and her joints have remained quiescent.

**Discussion/Results:**

Leishmania is an intracellular parasitic protozoan that is transmitted to humans by sandfly bites. It is endemic in Africa, America, Central Asia and the Mediterranean. It usually has an incubation period of 3-8 months. Those who are immunosuppressed, for example due to immunotherapy or HIV, are at increased risk of infection. The patient had only ever travelled to Portugal and Gibraltar. One trip to Gibraltar occurred whilst on dual immunosuppression with Infliximab and Methotrexate and prior to the development of neutropenia. She has no recollection of any insect bites.

Visceral leishmaniasis is variable in the severity of symptoms experienced ranging from asymptomatic to a febrile illness with lymphadenopathy, hepatospenomegaly, night sweats, cutaneous pigmentation, appetite loss and weight loss, which can be drastic. Our patient indeed described significant weight loss in the preceding year.

This case demonstrated the laboratory abnormalities which are typical of Visceral Leishmaniasis: pancytopenia and hypergammaglobulinaemia.

Treatment for visceral Leishmaniasis has classically been with pentavalent antimonials (based on the heavy metal antimony) such as Sodium stibogluconate. Their toxicity profile and growing resistance however has meant amphotericin B is often used first line. This is given intravenously, with the liposomal form preferred as it has lower toxicity. Our patient received 4 mg per kg daily on days 1–5, 10, 17, 24, 31, and 38, following the FDA-approved regimen for immunosuppressed patients.

Without treatment Leishmaniasis is usually fatal, with haemmorrhage, infection, cachexia and multiorgan failure as causes of death.

**Key learning points/Conclusion:**

Whilst patients with Rheumatological conditions may have cytopaenia due to their disease or its treatment, persistent, progressive or unexplained results should be investigated further. In patients with cytopaenia of unclear aetiology, thorough investigation with Haematology involvement is mandatory. You may find an unexpected diagnosis … we did!

